# Early Secreted Antigen ESAT-6 of *Mycobacterium tuberculosis* Promotes Protective T Helper 17 Cell Responses in a Toll-Like Receptor-2-dependent Manner

**DOI:** 10.1371/journal.ppat.1002378

**Published:** 2011-11-10

**Authors:** Samit Chatterjee, Ved Prakash Dwivedi, Yogesh Singh, Imran Siddiqui, Pawan Sharma, Luc Van Kaer, Debprasad Chattopadhyay, Gobardhan Das

**Affiliations:** 1 Immunology Group, International Centre for Genetic Engineering and Biotechnology, Aruna Asaf Ali Marg, New Delhi, India; 2 Department of Microbiology and Immunology, Vanderbilt University School of Medicine, Nashville, Tennessee, United States of America; 3 ICMR Virus Unit, Calcutta, ID & BG Hospital, GB 4, Beliaghata, Kolkata, India; Johns Hopkins School of Medicine, United States of America

## Abstract

Despite its relatively poor efficacy, Bacillus Calmette-Guérin (BCG) has been used as a tuberculosis (TB) vaccine since its development in 1921. BCG induces robust T helper 1 (Th1) immune responses but, for many individuals, this is not sufficient for host resistance against *Mycobacterium tuberculosis* (*M. tb*) infection. Here we provide evidence that early secreted antigenic target protein 6 (ESAT-6), expressed by the virulent *M. tb* strain H37Rv but not by BCG, promotes vaccine-enhancing Th17 cell responses. These activities of ESAT-6 were dependent on TLR-2/MyD88 signalling and involved IL-6 and TGF-β production by dendritic cells. Thus, animals that were previously infected with H37Rv or recombinant BCG containing the RD1 region (BCG::RD1) exhibited improved protection upon re-challenge with virulent H37Rv compared with mice previously infected with BCG or RD1-deficient H37Rv (H37RvΔRD1). However, TLR-2 knockout (TLR-2^-/-^) animals neither showed Th17 responses nor exhibited improved protection in response to immunization with H37Rv. Furthermore, H37Rv and BCG::RD1 infection had little effect on the expression of the anti-inflammatory microRNA-146a (miR146a) in dendritic cells (DCs), whereas BCG and H37RvΔRD1 profoundly induced its expression in DCs. Consistent with these findings, ESAT-6 had no effect on miR146a expression in uninfected DCs, but dramatically inhibited its upregulation in BCG-infected or LPS-treated DCs. Collectively, our findings indicate that, in addition to Th1 immunity induced by BCG, RD1/ESAT-6-induced Th17 immune responses are essential for optimal vaccine efficacy.

## Author Summary

Tuberculosis is a global health problem, with one-third of the global population infected with tubercle bacteria. Numerous studies have shown that Th1 cell responses are indispensable for protective immunity against TB. However, while the vaccine strain BCG induces sufficient Th1 cell response, this response does not appear to be sufficient for immune protection in many individuals. Here, we provide evidence for the first time that Th17 cell responses in the lung play a critical role for enhanced protection against TB. Surprisingly, the virulent *M. tb* strain H37Rv induced Th17 cell responses in the lung. Consequently, antibiotic-treated animals that were previously infected with H37Rv, as compared with similarly treated BCG-infected mice, generated improved protective immune responses against infection with virulent *M. tb*. We also provide evidence that the ESAT-6 protein, which is absent in BCG but present in H37Rv, induces IL-6 and TGF-β in dendritic cells in a TLR-2 and MyD88-dependent manner, which generates an environment that is conducive for the differentiation of Th17 cells in the lung. Our findings indicate that, in addition to Th1 cells, Th17 cells play a critical role in conferring optimal protection against TB.

## Introduction

Tuberculosis (TB) remains a major health problem, with an estimated one third of the world's population infected with *Mycobacterium tuberculosis,* the causative agent of TB, resulting in ∼3 million deaths annually. Bacillus Calmette-Guérin (BCG), the only TB vaccine presently used in humans, has been widely used throughout the world since its inception in 1921, and an estimated 3 billion people have received it [1]. However, its efficacy against pulmonary TB in adults is highly variable (0–80%) [2] and depends on ethnicity and geographical location [3], [4], [5]. The antigenic component(s) that is absent in BCG to elicit critical protective immune responses against TB has been an area of intense research [4], [5]. Early secreted antigenic target protein 6 (ESAT-6) is one of the most prominent antigens expressed by *Mycobacterium tuberculosis* (*M. tb*), but not by BCG [6], [7]. ESAT-6-specific T cells are frequently found in TB patients as well as in infected animals [8], [9], [10]. Thus, ESAT-6 is being extensively studied for its potential activity as a subunit vaccine [11]. T cell receptor transgenic T cells specific for ESAT-6 exhibit significant protection against TB [12]. Consistent with this, a recombinant BCG strain that contains region of difference 1 (RD1), which includes ESAT-6, exhibited improved protection against TB [13]. However, the basis of this improved protection remains elusive. Furthermore, the mechanism by which ESAT-6 vaccination induces protective immune responses against TB remains to be investigated.

Furthermore, deletion mutants of virulent *M. tb* strains for RD1 or ESAT-6 (a protein product of the RD1 region) resemble BCG in their infectivity and attenuation [14]. Therefore, these bacterial strains provide insight into the rational selection and design of suitable candidate vaccines for M. tb infection. It is clear that ***v***accination with BCG produces Th1 cell-mediated immune responses, and this is moderately effective in protecting against disseminated TB and against meningitis in children [15]. However, immune responses that are critical for protection against adult pulmonary TB remain incompletely understood. Recently, it has been shown that Th17 cell responses play an important role in establishing protective immune responses against TB [16]. However, Th17 cells do not contribute to the primary immune responses in tuberculosis infection [17]. The antigen-specificity of protective Th17 cell responses in *M. tb* vaccination has not been reported. The differentiation of Th17 cells involves the cytokines interleukin (IL)-6 and TGF-β [18], [19]. Earlier studies indicated that IL-6 production in DCs is regulated by microRNA-146a (miR146a) expression, which acts as a negative feedback regulator in TLR signalling by targeting IL-1R associated kinase (IRAK)-1 and TRAF6[20], [21]. miR146a inhibits the expression of IRAK-1 and TRAF6 and impairs NF-κB activity, which results in suppression of IL-6, IL-1β and TNF-α expression [21], [22]. Recently, it has been shown that expression of miR146a is also upregulated in viral and bacterial diseases to modulate immune responses [23], [24]. Therefore, we hypothesised that miR146a might have a key role in *M. tb* infection by regulating IL-6 production.

Here we show that H37Rv and recombinant BCG containing the RD1 region (BCG::RD1) induce improved vaccine efficacy compared with BCG and H37Rv deletion mutants for RD1 (H37RvΔRD1). The virulent strain H37Rv and BCG::RD1 induced both Th1 and Th17 cell responses, whereas BCG and H37RvΔRD1 induced only Th1 cell responses. Inhibition of IL-17 by neutralizing antibodies dramatically reduced the vaccine efficacy of H37Rv and BCG::RD1. H37Rv and BCG::RD1 induced IL-6 and TGF-β in DCs, which generated a microenvironment conducive to the differentiation of Th17 cells. In contrast, BCG and H37RvΔRD1 induced dramatically lower levels of IL-6 and TGF-β. Interestingly, production of both IL-6 and TGF-β in DCs induced by H37Rv and BCG::RD1 was dependent on the TLR-2/MyD88 signalling pathway. Furthermore, DCs infected with H37Rv or BCG::RD1 upregulated lower levels of miR146a compared with BCG and H37RvΔRD1, which differentially affected IL-6 production in infected DCs. Consistent with this, ESAT-6-treated DCs produced IL-6 and TGF-β in a TLR-2/MyD88-dependent manner, and facilitated the polarization of Th17 cell responses. miR146a expression in DCs was unaffected by ESAT-6 treatment and comparable to uninfected DCs, and ESAT-6 dramatically inhibited miR146a upregulation in BCG-infected or LPS-treated DCs. Therefore, these results indicate that interaction of ESAT-6 with TLR-2 generates a cytokine environment that facilitates the differentiation of Th17 cells, which in turn contributes to protection against TB.

## Results

### Virulent *M. tb* strain H37Rv induces Th17 cell responses in lung

**Figure 1:**
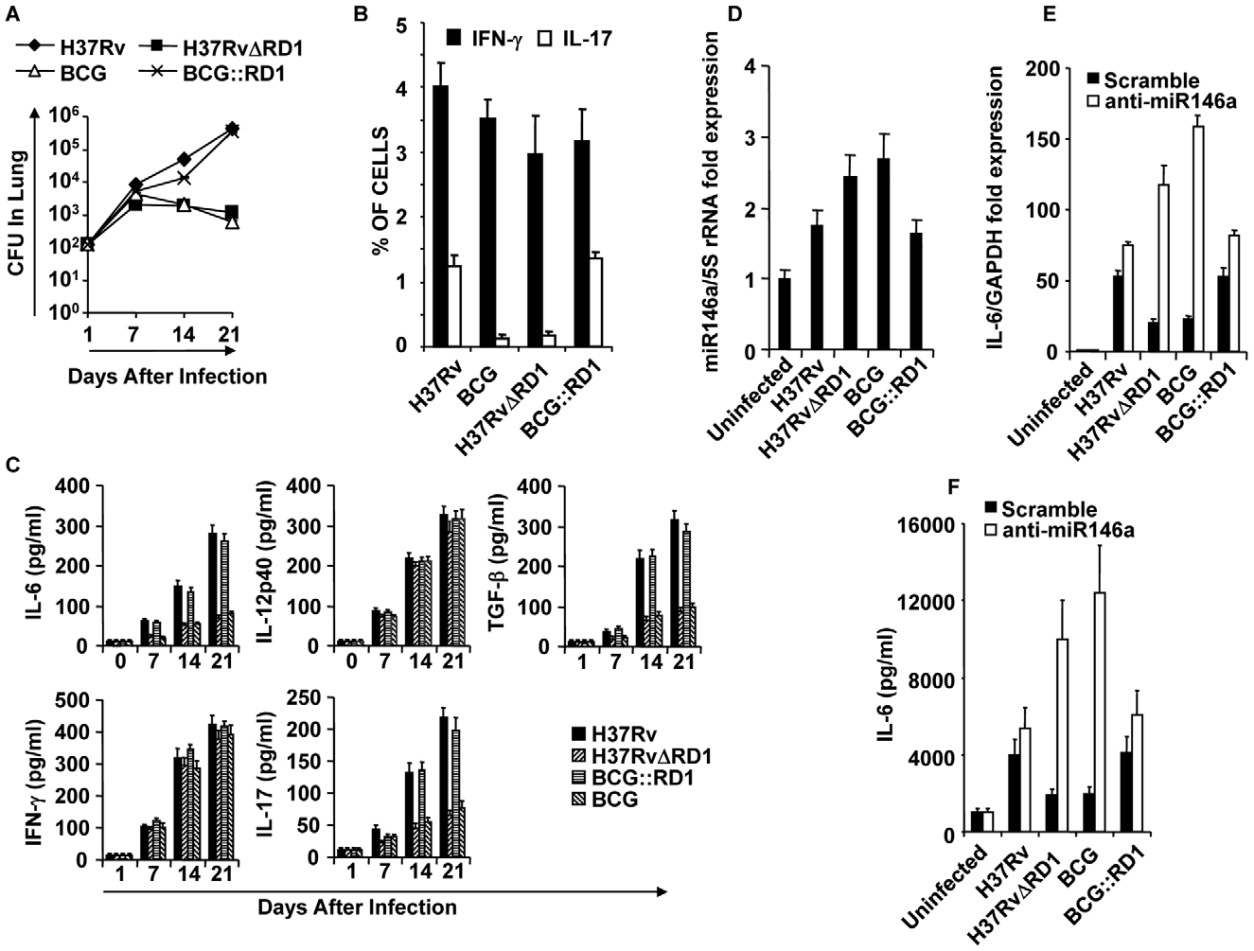
Infection with H37Rv or BCG::RD1 induces both Th1 and Th17 immunity, whereas BCG and H37RvΔRD1 selectively induce Th1 cell responses in the lung. C57BL/6 mice were challenged with H37Rv, BCG, H37RvΔRD1 or BCG::RD1 by the aerosol route and lungs were harvested at different time points. (**A**) CFU from the lung homogenate of mice that were infected with H37Rv, BCG, H37RvΔRD1 or BCG::RD1 strains. (**B**) Intracellular staining for IFN-γ and IL-17 of CD4^+^ T lymphocytes isolated from the lungs of infected mice. (**C**) Presence of IFN-γ and IL-17 in BAL fluid was measured by Luminex. (**D**) miR146a expression profile of DCs infected with different strains of bacteria. miR146a expression was normalized with 5S rRNA control primer. (**E**) IL-6 mRNA expression profile after infection of DCs with different bacterial strains and compared after knock-down of miR146a with anti-miR146a miRCURY LNA knock-down probes. IL-6 mRNA expression increases after knock-down of miR146a increases. (**F**) IL-6 cytokine production increases after knock-down of miR146a increases. The results shown are representative of at least 3–4 independent experiments.

It is well accepted that Th1 cell responses are indispensable for host protection against TB [25]. The vaccine strain BCG induces robust Th1 cell responses, yet it is not an effective vaccine against adult pulmonary TB in many individuals [3], [4], [5]. Therefore, additional immune response(s) are required for optimal vaccine efficacy. Recently, Th17 cells have been implicated in protective immunity against TB [16]. Previous studies have demonstrated that RD1, which is absent in BCG, plays a dominant role in protective immune responses and bacterial virulence [14]. Thus, an RD1 deletion mutant of H37Rv resembles BCG in its infectivity [7]. Therefore, we tested the virulence and cytokine production by virulent strain H37Rv and vaccine strain BCG. We challenged C57BL/6 mice with a low dose (∼110 CFU) of H37Rv or BCG by the aerosol route. We found that H37Rv and BCG replicated to a similar extent during the initial phase of the infection (**Fig. 1A**). However, at later time points, growth of BCG was gradually diminished (p<0.032) (**Fig. 1A**), suggesting that adaptive immune responses play an important role in clearing BCG. After 21 days of infection, only a few bacilli were found in the lungs of animals infected with BCG (**Fig. 1A**). These kinetics of BCG and H37Rv infection are in agreement with the published literature [25], [26], [27], [28]. Interestingly, we observed significantly higher numbers of IL-17-producing CD4^+^ T cells in the lungs of animals infected with H37Rv, as compared with BCG (p<0.0001) **(** **Fig. 1B** **)**. In sharp contrast, both H37Rv and BCG induced IFN-γ in the bronchoalveolar lavage (BAL) fluid **(** **Fig. 1B & C** **)**. This is further supported by the increased amounts of IL-17 produced in the BAL fluid of mice infected with H37Rv but not BCG (p<0.0001) **(** **Fig. 1B & C** **)**. Although significantly lower, BCG-infected animals produced some IL-17 in the BAL fluid **(** **Fig. 1C** **)**. However, we were unable to detect IL-17-producing CD4***^+^*** cells in the lung of BCG-infected animals **(** **Fig. 1B** **)**, suggesting that the source of IL-17 in BCG-infected animals is not Th17 cells. It has been previously shown that γΔ T cells are the primary source of IL-17 in the lung during BCG infection [29].

Consistent with the observation that H37Rv induces higher Th17 responses in the lung, we found significant levels of IL-6 (p<0.001***)*** and TGF-β (p<0.001), two key cytokines required for the differentiation of Th17 cells [18], [19], in the BAL fluid of animals infected with H37Rv **(** **Fig. 1C** **)**. In contrast, both H37Rv and BCG produced similar amounts of IL-12p40 **(** **Fig. 1C** **)**, a cytokine that supports Th1 cell differentiation. These observations suggested that H37Rv creates an environment that is conducive to the differentiation of both Th1 and Th17 cells, whereas BCG promotes Th1 cell differentiation only. To investigate the molecular basis for the capacity of H37Rv to induce high levels of IL-6, we compared induction of microRNA-146a (miR146a), a negative regulator of innate immune components such as IL-6, in infected DCs[20], [21]. Interestingly, we found that BCG significantly upregulated miR146a in DCs as compared with H37Rv-infected DCs (p<0.01) **(** **Fig. 1D** **)**. Furthermore, specific knock-down of miR146a expression dramatically upregulate both mRNA and protein level of IL-6 in BCG-infected DCs **(** **Fig. 1E & F** **)**. These data indicated that H37Rv promotes the differentiation of both Th1 and Th17 cell responses, whereas BCG induces Th1 responses but fails to support Th17 cell differentiation due to its induction of miR146a in infected cells. Nonetheless, a recent study indicated that BCG is unable to induce IL-17-producing cells during primary challenge but can do so after several rounds of challenge [**30**]. Therefore, it is highly likely that repeated immunization with BCG induces IL-17 production by innate-like cells or recruits IL-17-producing cells to the lung due to the local chemokine milieu.

Compared with virulent H37Rv, BCG possesses multiple deletion mutations. These mutations are called regions of difference (RD). Among these RD regions, RD1 is the most dominant and plays an important role in virulence [31]. Thus, an RD1 deletion mutant of H37Rv resembles BCG in its infectivity [14]. Within the RD1 region, the proteins ESAT-6 and CFP-10 have been shown to form a complex and participate in a type VII secretion system [6], [32]. Therefore, we tested the virulence and cytokine production induced by H37RvΔRD1. Consistent with the results obtained for BCG, we observed that H37RvΔRD1 initially grew to a similar extent as the parental H37Rv strain, but at later time points its growth gradually diminished (**Fig. 1A**). Akin to BCG, H37RvΔRD1 failed to induce IL-17 production in the lung (**Fig. 1B & C**). However, both strains induced similar quantities of IFN-γ **(** **Fig. 1B & C** **).** Furthermore, IL-12 production was comparable between these strains, whereas IL-6 and TGF-β production was dramatically reduced as compared with H37Rv **(** **Fig. 1C** **)**. To provide further support for these findings, we performed similar experiments with the BCG recombinant strain in which the RD1 region was reintroduced (BCG::RD1). In agreement with previous reports [33], BCG::RD1 showed a dramatically higher virulence as compared with the parental BCG strain, but was comparable to H37Rv **(** **Fig. 1A** **)**. Consistent with this finding, BCG::RD1 induced both IFN-γ and IL-17 **(** **Fig. 1** **B & C)**, as well as the Th1- and Th17-differentiating cytokines IL-12p40, IL-6, and TGF-β **(** **Fig. 1C** **)** in the lungs. Additionally, H37RvΔRD1 induced significantly higher levels of miR146a than the parental H37Rv strain in infected DCs (p<0.02) **(** **Fig. 1D** **)**, and knock-down of miR146a significantly improved IL-6 production both at mRNA transcript and protein level **(** **Fig. 1E & F** **)**. To provide further support for these data, we performed similar experiments with the BCG recombinant strain containing RD1 (BCG::RD1). Consistently, BCG::RD1 induced both IFN-γ and IL-17 **(** **Fig. 1** **B & C)**, and the Th1- and Th17-differentiating cytokines IL-12p40, IL-6, and TGF-β **(** **Fig. 1C** **)** in the lungs, but failed to induce miR146a in infected DCs **(** **Fig. 1D** **)**. These observations suggested that the RD1 region is responsible for the induction of Th17 cell responses.

### IL-17 induced by H37Rv or BCG::RD1 mediates improved vaccine efficacy

**Figure 2:**
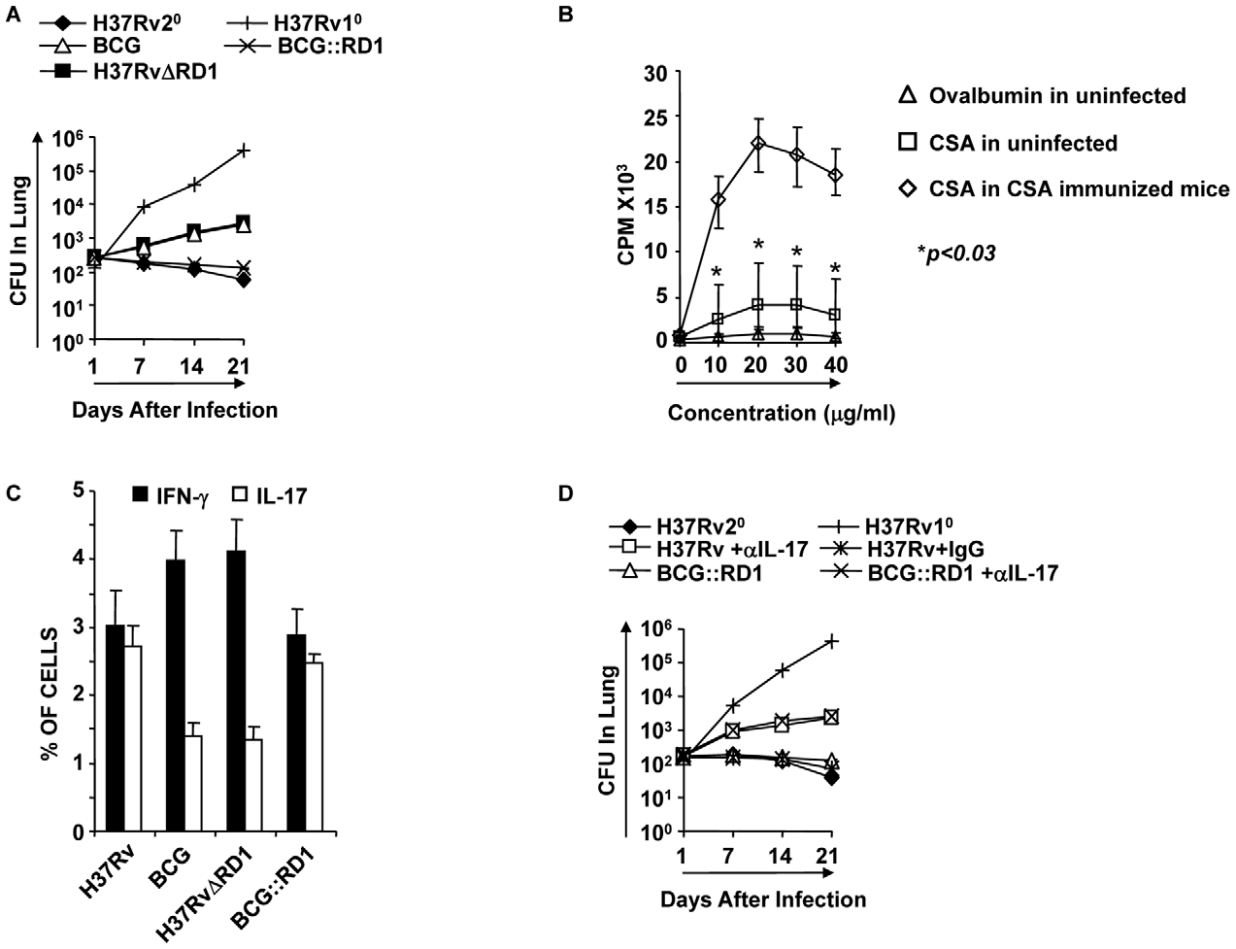
Clearance of H37Rv and BCG::RD1 induces improved protective immune responses compared with BCG and H37RvΔRD1. Animals were infected with the indicated bacterial strains for 30 days and treated with antibiotics for an additional 28 days. Animals were rested for one month and re-challenged with a low dose of H37Rv (∼110 bacilli) by aerosol challenge. Some groups were injected with anti-IL-17 antibodies (100 µg/mouse intravenous) or mouse IgG every 72 hrs until sacrifice at the time points indicated. (**A**) CFU in the lung homogenates of C57BL/6 mice that were previously infected with H37Rv, BCG, H37RvΔRD1 or BCG::RD1 strains. (**B**) Splenocytes were isolated from sixteen randomly selected animals from our colony or from CSA-immunized mice. Proliferation of splenocytes in response to CSA was measured by [^3^H]-thymidine incorporation assay. As a negative control, spleen cells were stimulated with OVA, and as a positive control, we measured the response against CSA by splenocytes from CSA-immunized mice. (**C**) Intracellular staining of CD4^+^ T lymphocytes in the lung of mice re-challenged with H37Rv. (**D**) CFU in lung homogenates of mice that received anti-IL-17 or control antibodies. Results presented in (A), (C) and (D) are representative of three independent experiments.

Previously, it has been shown that IL-17 plays an important role in the secondary immune response following vaccination with virulent H37Rv [16]. Thus, we examined whether IL-17 induced by virulent strains contributes to improved vaccine efficacy. For this purpose, we infected animals with H37Rv, BCG, H37RvΔRD1, or BCG::RD1 for 30 days. These animals were subsequently treated with antibiotics for four weeks, and then rested for an additional month. We could not find any detectable *M. tb* organisms in these animals. These mice were then challenged with H37Rv through aerosol infection. We found that animals previously infected with BCG or H37RvΔRD1 exhibited robust protective immunity compared with primary infection (p<0.01) (**Fig. 2A**). Interestingly, we found that animals that were previously infected with H37Rv produced dramatically enhanced protective immune responses (**Fig. 2A**). This is in agreement with previous reports suggesting that virulent strains of *M. tb* H37Rv induce superior protective immune responses [34], [35]. However, the kinetics of host protective responses in our hand are somewhat different from these studies, which may be due to differential environmental factors in different geographical regions. In fact, it has been well documented that the efficacy of BCG in human vaccine trials dramatically varies depending on the geographical location ([3], [4], [5]). Nevertheless, we tested M. tb antigen-specific responses induced by randomly selected animals from our colony. We challenged splenocytes from sixteen animals with M. tb-derived complete soluble antigen (CSA) or the unrelated antigen ovalbumin (OVA) and measured lymphoproliferation. We observed that animals from our colony responded weakly to CSA, whereas no response was detected against OVA. Therefore, these animals were likely exposed to environmental organism(s) that share antigenic similarities with M. tb. As a positive control, we used spleen cells from CSA immunized mice, which showed dramatic proliferation against in vitro rechallenge with CSA (Fig 2B). These observations might also be relevant to the variable vaccine response of BCG. In either case, our findings suggested that, while BCG and H37RvΔRD1 induced significant protective immunity against TB, this was not sufficient to confer complete protection against disease pathology. In contrast, H37Rv and BCG::RD1 induced improved protective immune responses. Collectively, these observations suggested that the RD1 region enhances protective immune responses. Importantly, we found that animals that were previously infected with H37Rv or BCG::RD1 induced significantly higher numbers of Th17 cells in the lungs than animals infected with BCG or H37RvΔRD1 (p<0.001) **(** **Fig. 2C** **)**. Therefore, we tested whether IL-17 was responsible for the improved vaccine efficacy of H37Rv or BCG::RD1. For this purpose, we injected animals with anti-IL-17 or control mouse IgG antibodies every 72 hours during re-challenge with H37Rv or BCG::RD1. Treatment with anti-IL-17 abrogated the observed enhancement in protective immune responses induced by H37Rv or BCG::RD1 **(** **Fig. 2D** **)**. Therefore, these observations suggested that H37Rv induced Th17 cell responses, which complemented Th1 cell responses for improved protection against TB.

### Dendritic cells infected with H37Rv or BCG::RD1 direct Th1 and Th17 cell responses, whereas BCG and H37RvΔRD1 selectively induce Th1 cell responses

**Figure 3:**
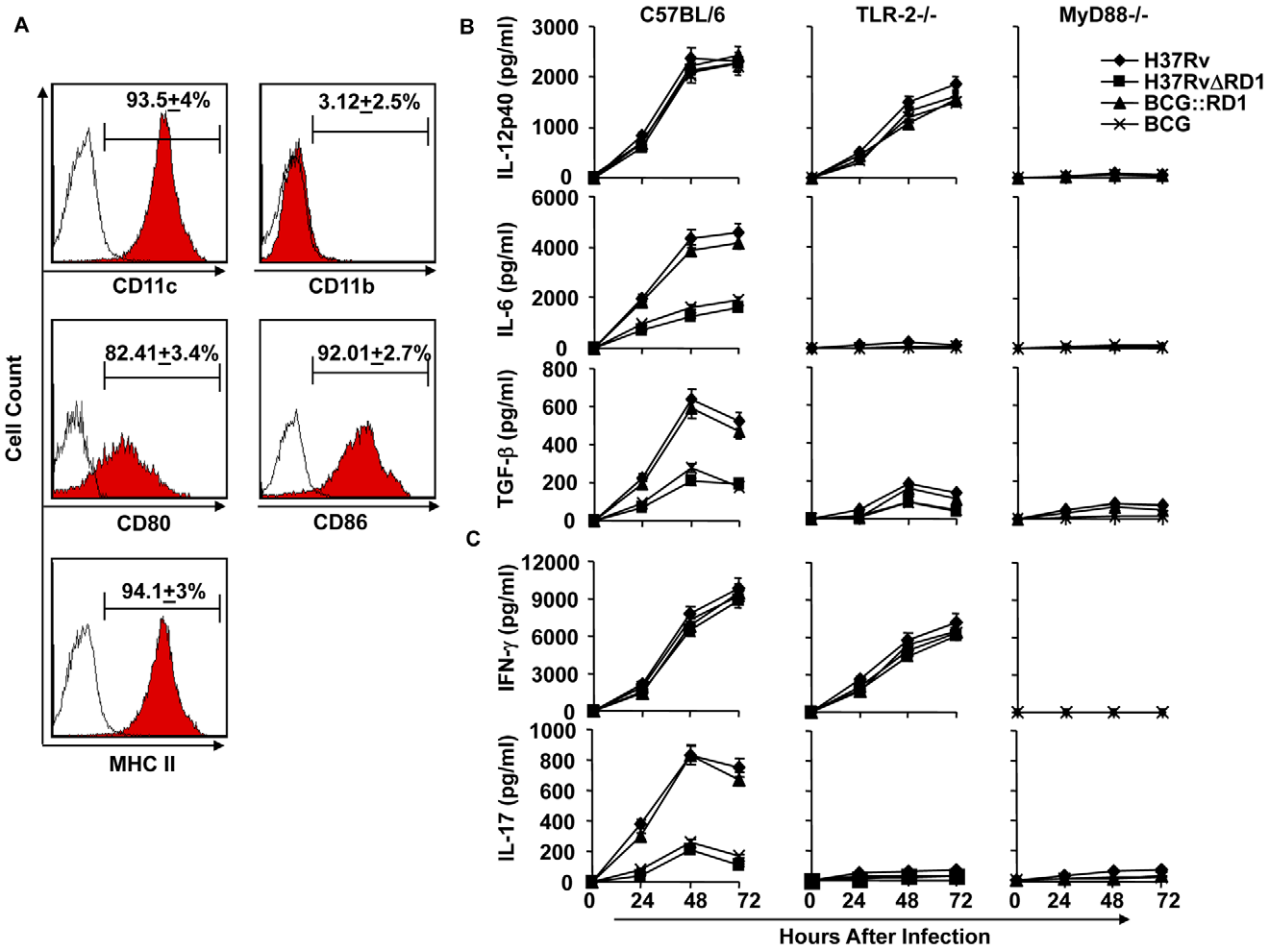
H37Rv directs differentiation of both Th1 and Th17 cells by inducing IL-12p40, IL-6, and TGF-β in DCs. (**A**) Characterization of dendritic cells (DCs) by flocytometric analysis using anti-CD11c, CD11b, -CD80, -CD86, -MHC class II, and - IgG2a(isotype control). (**B**) Production of IL-12p40, IL-6, and TGF-β in DCs infected with H37Rv, BCG, H37RvΔRD1 or BCG::RD1. (**C**) Induction of IFN-γ and IL-17 in the culture supernatants of OT-II TCR Tg CD4^+^ T cells co-cultured with infected DCs Results presented here are representative of at least three independent experiments.

**Figure 4:**
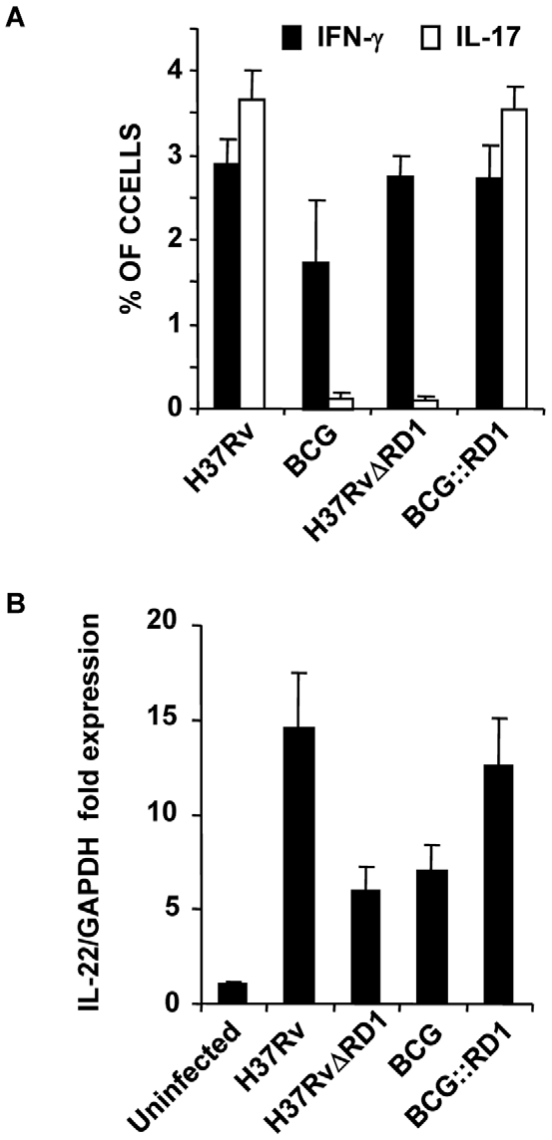
H37Rv directs differentiation of both Th1 and Th17 cells. (**A**) Intracellular cytokine staining for IFN-γ and IL-17 in OT-II TCR Tg CD4^+^ T cells co-cultured with infected DCs. (**B**) IL-22 mRNA expression profile from OT-II TCR Tg CD4^+^ T cells co-cultured with DCs after infection of DCs with H37Rv, BCG, BCG::RD1 or H37RvΔRD1. Results are representative of at least three independent experiments.

Our *in vivo* experiments demonstrated that H37Rv induces Th17 cell differentiation. Therefore, to provide insight into the mechanism whereby H37Rv promotes Th1 and Th17 cell differentiation, we compared the cytokines induced by DCs (characterized with CD11c, CD11b, CD80, CD86, and MHC Class II markers **Fig. 3A**) infected with H37Rv, BCG::RD1, BCG, and H37RvΔRD1. We found that H37Rv- or BCG::RD1-infected DCs produced substantial amounts of IL-12p40, IL-6, and TGF-β (**Fig. 3B**). However, BCG and H37RvΔRD1 induced dramatically reduced amounts of IL-6 (p<0.001) and TGF-β (p<0.001) by DCs than H37Rv and BCG::RD1 (**Fig. 3B**). Nevertheless, IL-12p40 was induced at comparable levels by all bacterial strains. Interestingly, we found that IL-6 and TGF-β production was dependent on TLR-2 and MyD88, whereas IL-12p40 production was independent of TLR-2 but required MyD88 (**Fig. 3B**). To determine whether these cytokines play a role in Th cell differentiation, we co-cultured ovalbumin (OVA)-specific CD4^+^ T cells from OT-II T cell receptor (TCR) transgenic (Tg) animals with infected DCs in the presence of OVA peptide and collected supernatant to determine the production of IFN-γ and IL-17 (**Fig. 3C**). The results, which were supported by intracellular cytokine staining, indicated that H37Rv-infected DCs directed the differentiation of both IL-17- and IFN-γ-producing cells (**Fig. 3C** **&** **4A**). In sharp contrast, DCs infected with BCG or H37RvΔRD1 supported only Th1 cell differentiation. While the levels of IFN-γ production were similar for DCs infected with H37Rv, BCG, H37RvΔRD1 or BCG::RD, production of IL-17 was significantly higher in DCs infected with H37Rv or BCG::RD1 as compared with cells infected with BCG or H37RvΔRD1 (p<0.001). Furthermore, it is known that IL-22 is also secreted by IL-17 producing Th cells and recent study suggested that IL-22 was upregulated during M. tb infection in rhesus macaques and protective in function[36]. Therefore, we have also checked the IL-22 mRNA transcript level in our DC-T cells co-culture experiments and found that IL-22 mRNA transcript was 5–8 fold upregulated in H37RV or BCG::RD virulent strains compared to BCG and H37RvΔRD1 **(** **Fig. 4B** **)**. Therefore, these data indicated that the RD1 locus plays an important role in directing Th17 cell responses during *M. tb* infection.

### ESAT-6 drives Th17 cell differentiation by inducing IL-6 and TGF-β production in DCs

**Figure 5:**
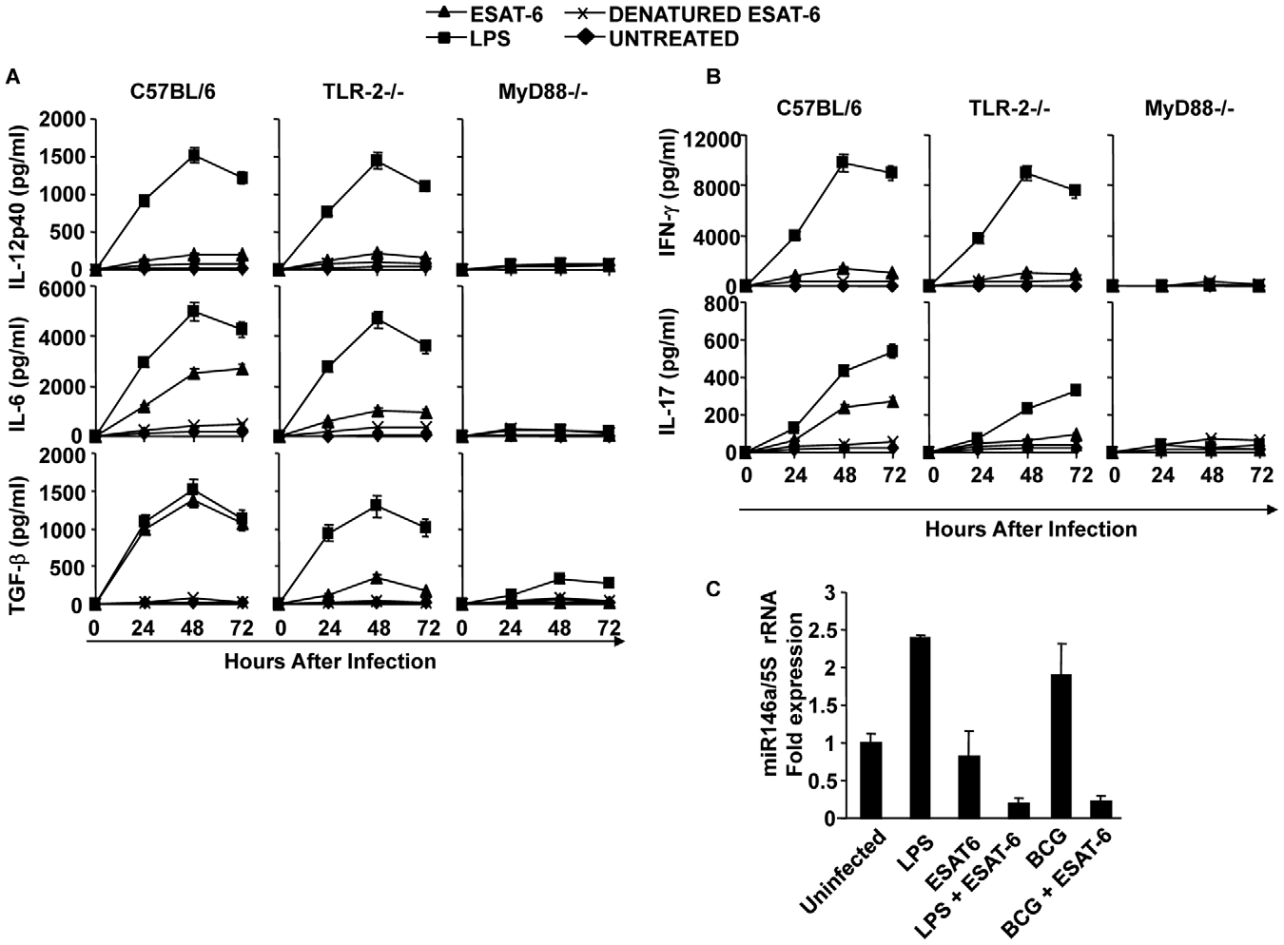
ESAT-6 induces IL-6 and TGF-β in DCs in a TLR-2- and MyD88-dependent manner. DCs from wt, TLR-2^-/-^, or MyD88^-/-^ mice were treated with ESAT-6 protein, LPS, or denatured ESAT-6 protein, or untreated. Supernatants were harvested at different time points and cytokines were measured. (**A**) IL-12p40, IL-6, and TGF-β in supernatants of the cultured DCs. (**B**) Production of IFN-γ and IL-17 by OT-II TCR Tg CD4^+^ T cells co-cultured with DCs that were treated with ESAT-6 protein and LPS. (**C**) Quantitative expression of miR146a profile after LPS and ESAT-6 treatment in DCs. Results presented in (A) and (B) are representative of six independent experiments and results presented in (C) are representative of three independent experiments.

ESAT-6-reactive T cells are prevalent in TB patients and in animals infected with *M. tb* [8], [9], [10]. Furthermore, it has been shown that ESAT-6-specific T cells provide substantial protection against TB [12]. Therefore, it has been assumed that ESAT-6 is a good candidate for development of a TB vaccine [37]. From the preceding section, it was clear that the RD1 region plays an important role in directing Th17 cell differentiation, which in turn contributes to protective immune responses against TB. Differentiation of Th17 cells requires IL-6 and TGF-β simultaneously [18], [19]. Therefore, we determined whether ESAT-6 induces these cytokines in DCs. We found that DCs treated with ESAT-6 produced both IL-6 and TGF-β (**Fig. 5A**). However, ESAT-6 did not induce IL-12 in DCs (**Fig. 5A**). CD4^+^ T cells from OT-II TCR Tg mice co-cultured with DCs in the presence of ESAT-6 and OVA differentiated into IL-17-producing cells (**Fig. 5B**).

### TLR-2 is required for the induction of IL-6 and TGF-β in DCs by ESAT-6

Our findings indicated that ESAT-6 induces IL-6 and TGF-β production in DCs, which drives Th17 cell differentiation. Previous reports have suggested that ESAT-6 binds to TLR-2 [38]. Therefore, we tested whether TLR-2 is required for the capacity of ESAT-6 to induce IL-6 and TGF-β production. For this purpose, we compared cytokine production by DCs derived from wild type and TLR-2^-/-^ mice. We found that DCs from TLR-2^-/-^ mice were unable to produce IL-6 and TGF-β (**Fig. 5A**). To confirm that innate immune signalling is required for the capacity of ESAT-6 to induce IL-6 and TGF-β production in DCs, we performed experiments with DCs isolated from MyD88^-/-^ mice. As expected, DCs from MyD88^-/-^ mice were also unable to produce IL-6 and TGF-β in response to ESAT-6 stimulation (**Fig. 5A**). Interestingly, we observed that ESAT-6 dramatically inhibited miR146a expression in both LPS- and BCG-treated DCs **(** **Fig. 5C** **)**. Thus, ESAT-6 allows IL-6 production in DCs by inhibiting the induction of miR146a. Taken together, our findings indicated that IL-6 and TGF-β induced by ESAT-6 in DCs generate an environment that promotes the differentiation of Th17 cells.

Next, we determined the capacity of DCs treated with H37RvΔRD1 to produce IL-6 and TGF-β. Our results clearly showed that neither H37RvΔRD1 nor BCG were able to induce IL-6 and TGF-β in DCs derived from either wild type, TLR2^-/-^, or MyD88^-/-^ mice (**Fig. 3B**). Interestingly, we found that the parental strain H37Rv, BCG::RD1, H37RvΔRD1 and BCG induced IL-12p40 production in DCs isolated from both wild type and TLR-2^-/-^ mice. However, none of these strains induced IL-12p40 production in DCs derived from MyD88^-/-^ mice. Therefore, taken together, these observations suggested that ESAT-6 induces IL-6 and TGF-β production in a TLR-2-dependent manner. In contrast, production of IL-12p40 by DCs following infection with mycobacteria is independent of ESAT-6 and TLR-2 expression. However, IL-12p40 production induced by mycobacteria is dependent on MyD88 signalling.

### TLR-2^-/-^ animals fail to induce *M. tb*-mediated Th17 cell responses and show enhanced susceptibility to *M. tb* infection

**Figure 6:**
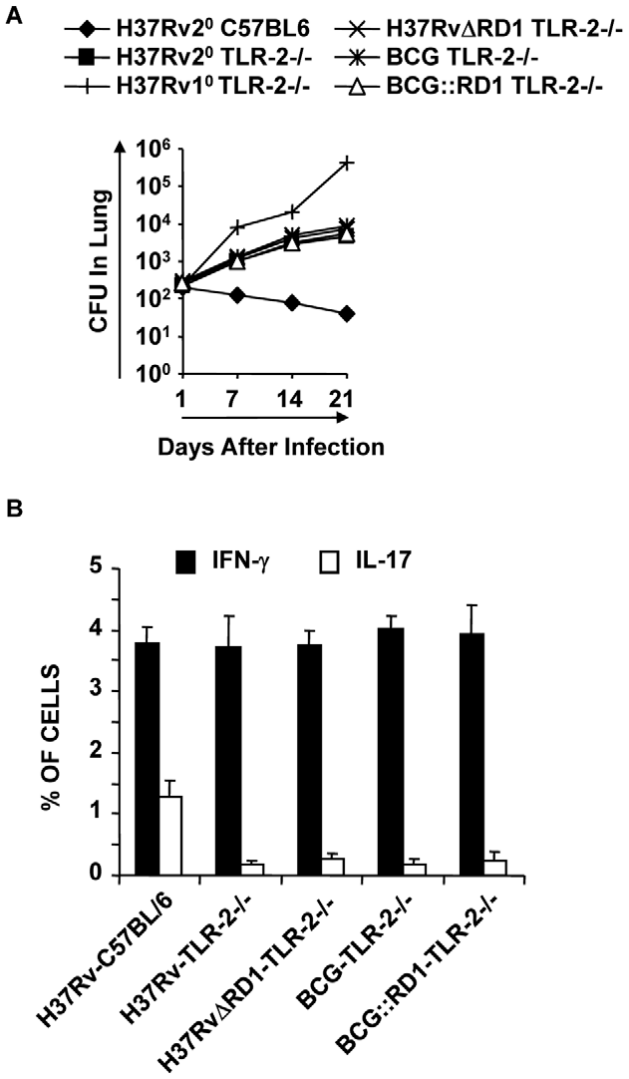
TLR-2-deficient animals induce reduced levels of protective immune responses. For *in vivo* experiments, wild type and TLR-2^-/-^ mice were infected with H37Rv for 30 days, and subsequently treated with antibiotics for 4 weeks as described in the materials and methods. Mice were then re-challenged with H37Rv. Groups of animals were sacrificed for the following studies. (**A**) CFU in lung homogenates of mice re-challenged with H37Rv. (**B**) Intracellular cytokines in CD4^+^ T lymphocytes derived from the lung. Results presented here are representative of three independent experiments.

From the preceding section it is clear that interaction of ESAT-6 with TLR-2 creates an environment that is conducive to the differentiation of Th17 cells, which in turn results in protective immunity against TB. Therefore, to confirm that TLR-2 signalling is important for the observed Th17 cell responses and improved vaccine efficacy, we performed vaccination experiments in TLR-2^-/-^ animals. These animals were infected with H37Rv and subsequently treated with antibiotics as described in the materials and methods. These animals were then challenged with virulent strain H37Rv. H37Rv-immunized TLR-2^-/-^ mice generated protective immunity against H37Rv at a level similar to wild-type mice immunized with BCG during primary challenge (**Fig. 6A**). However, TLR-2^-/-^ mice exhibited reduced protective immune responses as compared with wild type littermates (**Fig. 6A**). H37RvΔRD1 and BCG::RD1 also showed reduced protection in TLR-2^-/-^ mice **(** **Fig. 6A** **)** similar to BCG and H37Rv. Other mycobacterial components, such as LAM and lipoproteins, can also activate TLR-2 [39]. Therefore, the observed differences in protective immune responses in TLR-2^-/-^ animals could be caused by multiple TLR-2-dependent agonists. However, most of the *M. tb*-derived TLR-2 ligands induce only suppressive immune responses [39]. Therefore, the observed protective responses are most likely contributed by RD1-derived proteins. Furthermore, these differences are comparable with responses induced by H37Rv versus BCG in wild-type animals.

Next, we analyzed effector T cells in the lungs. We found that TLR-2^-/-^ animals generated IFN-γ-producing cells comparable to wild type littermates. However, these animals produced significantly fewer numbers of IL-17-producing cells in their lungs (p<0.001) **(** **Fig. 6B** **)**. This finding is further strengthened by a recently published report, suggesting that TLR-2 is indispensible for the generation of Th17 responses during *M. tb* infection [40]. Therefore, these observations suggested that TLR-2 plays an important role in mounting Th17 cell responses to H37Rv, which in turn confers protective immunity to TB.

## Discussion

It is well accepted that Th1 cells play a central role for protection against TB [41]. Therefore, animals that are deficient in IFN-γ, IFN-γ receptor, Stat-4, T-bet, or IL-12 exhibit increased susceptibility to *M. tb* infection [25], [42], [43]. BCG induces a robust Th1 response, but this does not appear to be sufficient for optimal protection against challenge with virulent *M. tb* [44]. Abundant Th1 cell responses have been found in TB patients as well as *M. tb* infected animals [45], [46]. Thus, Th1 cells alone are not sufficient for protection against TB. Therefore, in addition to Th1 cell responses, a vaccine needs to induce additional Th cell response(s) to provide optimal protection against TB. Recently, it has been shown that Th17 cells play an important role in the secondary immune response against TB [16]. However, Th17 cells do not appear to participate in the primary immune response against TB [17]. Our findings clearly demonstrated that BCG and H37RvΔRD1 are unable to induce Th17 cell responses in the lung. Therefore, we considered the possibility that BCG lacks an antigen that drives Th17 cell differentiation and that such a response is required for optimal protection against TB. We observed that the virulent *M. tb* strain H37Rv and the engineered BCG::RD1 strain induced Th17 cell responses, which correlated with improved protection against re-infection and, thus, improved vaccine efficacy. This is in agreement with a previous report indicating that immunization with virulent *M. tb* H37Rv induces superior protective memory T cell responses [34]. However, unlike our results these authors found only 2–3 log differences in CFUs upon re-challenge with virulent H37Rv. This apparent discordance could be due to several differences in the experimental procedures employed. Jung et al. (2005) employed an extended (100 days) antibiotic treatment protocol, which may have influenced immune responses, and increased the age of the mice at re-challenge. Furthermore, these investigators rechallenged mice with a higher (two-fold) dose of bacteria. In addition, it is also possible that differences in the microflora in different animal facilities might contribute to these apparent differences. Indeed, our results demonstrated that unimmunized animals from our facility were able to respond to M. tb antigens, albeit weakly, suggesting that exposure to environmental organisms that share antigenic properties with M. tb might contribute to improved protection. This may explain the differential vaccine responses against BCG that have been observed in different geographical locations and in subjects from different ethnicities.

Previous reports have suggested that ESAT-6, a protein within the RD1 region that is absent in BCG, is a promising vaccine candidate [8]. Furthermore, deletion mutants of H37Rv for RD1 resembled BCG in many aspects [8]. Interestingly, we found that the RD1 mutant of virulent H37Rv was unable to induce Th17 cell responses. BCG and H37RvΔRD1 were unable to induce persistent infection and, hence, the bacterial load declined much more rapidly [14] over time as compared with H37Rv or BCG::RD1. Therefore, the observed differential Th1 and Th17 cell responses could be related to bacterial replication and antigenic load. However, previous reports indicated that BCG inhibits Th17 cell responses in lung and other organs [47], [48]. Furthermore, primary infection with a high dose (2.5×10^5^ CFU) of BCG by intratracheal injection was unable to induce IL-17 in the lung, until re-challenge with PPD-coated beads, and the cellular source of IL-17 produced in this situation is not known [49]. Therefore, BCG alone, even at a high dose, is unable to induce Th17 cell responses. Nonetheless, a recent report indicated that BCG was unable to induce IL-17-producing cells in a primary challenge, however it did so upon repeated re-challenge [30]. Although our study indicated that the RD1 recombinant strain exhibited an improved vaccine efficacy compared with the parental BCG strain, it does not exclude a role for other RD regions in inducing improved host protective immune responses. We found that H37Rv and BCG::RD1 induce both Th1 and Th17 cell responses that contribute to improved vaccine efficacy as compared with BCG, which selectively induces Th1 cell responses. Th17 cell responses are directed by IL-6 and TGF-β, derived from antigen presenting cells (APCs). Therefore, pathogen-associated molecular patterns (PAMP) encoded within the RD1 region are likely responsible for inducing these two cytokines. Considering that ESAT-6 induces protective immune responses and that the RD1 mutant was unable to induce Th17 cell responses, we considered the possibility that ESAT-6 induces Th17 cell-polarizing cytokines in APCs. Therefore, we tested IL-6 and TGF-β production in DCs infected with the wild-type and RD1 mutant H37Rv strain. These studies revealed that the mutant strain induced significantly lower levels of IL-6 and TGF-β in DCs. Furthermore, our studies with miR146a, a negative regulator of innate immune components such as IL-6 in infected DCs [21], suggested that BCG and H37RvΔRD1 significantly upregulated miR146a in DCs as compared with H37Rv- or BCG::RD1-infected DCs or uninfected DCs. Consistent with these findings, knock-down of miR146a expression dramatically upregulated IL-6 production in BCG-infected DCs. Of note, however, our studies cannot exclude the possibility that, in addition to ESAT-6, other factors encoded within the RD1 region contribute to the enhanced vaccine efficacy of BCG::RD1.

Previous studies have provided evidence that ESAT-6 directly binds to toll-like receptor-2 (TLR-2), a pattern recognition receptor (PRR) [38]. This led us to investigate the capacity of DCs from TLR-2^-/-^ animals to produce Th17 cell-polarizing cytokines in response to ESAT-6 treatment. Indeed, we found that production of IL-6 and TGF-β was dependent on TLR-2. To further substantiate this observation, we performed experiments with DCs from MyD88^-/-^ animals. Consistent with the results obtained with TLR-2^-/-^ mice, DCs from MyD88^-/-^ mice were unable to produce IL-6 and TGF-β in response to ESAT-6 treatment. However, a previous report suggested that engagement of TLR-2 on macrophages by ESAT-6 inhibits LPS-induced cytokine production, especially IL-6 and IL-12p40 [38]. Therefore, we revisited this issue for DCs. In our hands, we did not observe any influence of ESAT-6 on IL-12 production by DCs. However, ESAT-6 augmented IL-6 and TGF-β production by LPS-stimulated DCs. While the reasons for these discordant findings remain unknown, we speculate that the cell line used in the experiments by Pathak et al. [38] might have already been tolerized by LPS, so that secondary stimulation with ESAT-6 resulted in even lower production of IL-6 due to higher steady-state miR146a levels. [22].

It has been well-established that ESAT-6-specific T cells are prevalent in TB patients and in animal models of TB [8], [9], [10]. Furthermore, ESAT-6-reactive TCR Tg cells confer substantial protection against TB in an animal model [12]. In fact, it has been shown that ESAT-6 recombinant BCG provides protection against TB [13]. However, the mechanism of ESAT-6-mediated protection has been unclear until now.

One difference between mouse models and human infection with *M. tb* is that wild-type *M. tb* does not offer significant protection against reinfection in humans, despite containing ESAT-6 and other major antigens. This could be due to various reasons. First, there might be genetic differences between mice and humans that cause altered immune responses. Second, environmental exposures may alter protective immunity. Third, different individuals might respond differently to drugs used to treat TB and, thus, it is difficult to determine whether treatment was complete, while the remaining bacteria may cause secondary infection. Fourth, *M. tb* evolved many different types of immune evasion mechanisms [50]. For example, we have recently shown that bacteria that are within granuloma-like structures are sequestered from host protective immune responses by mesenchymal stem cells [51].

In summary, our findings indicate that, in addition to Th1 cells, Th17 cells play a critical role in conferring optimal protection against TB. The ESAT-6 protein, which is present in H37Rv and BCG::RD1 but not in BCG and H37RvΔRD1, directs Th17 cell differentiation by inducing IL-6 and TGF-β in DCs in a TLR-2- and MyD88-dependent manner. Therefore, ESAT-6 can contribute to vaccine preparations by promoting Th17 cell responses.

## Materials and Methods

### Ethics statement

Animal experiments were performed according to the guidelines approved by the Institutional Animals Ethics Committee meeting held on 22^nd^ November 2007 at ICGEB (approval ID; ICGEB/IAEC/IMM-13/2007), New Delhi, India and Department of Biotechnology (DBT) guidelines, Government of India. All mice used for experiments were ethically sacrificed by asphyxiation in carbon dioxide according to institutional and DBT regulations.

### Mice

C57BL/6 and OT-II TCR transgenic mice (6–8 wks of age) were initially purchased from The Jackson Laboratories, USA. TLR-2 and MyD88 knock-out mice (6–8 weeks of age), both on a C57BL/6 background, were the kind gift of Prof. Ruslan Medzhitov, Yale University, New Haven, USA. All animals were subsequently bred and maintained in the animal facility of the International Centre for Genetic Engineering and Biotechnology (ICGEB), New Delhi, India.

### Bacteria

*Mycobacterium tuberculosis* strain H37Rv was a kind gift from the Colorado State University repository. H37RvΔRD1 and BCG were a kind gift from Prof. David Sherman, (SBRI, Seattle, WA,USA). The integrative cosmid vector pYUB412 (control vector) and the recombinant cosmid vector RD1-2F9 harboring RD1 locus of M. tuberculosis [33] were kind gifts from Prof. Stewart Cole of the École Polytechnique Fédérale de Lausanne (EPFL), Switzerland. The control and recombinant cosmids were electroporated individually into electrocompetent cells of BCG (Danish) to obtain BCG::YUB412 and BCG::RD1 strains, essentially as described previously [33]. Briefly, 100 ml of bacilli suspension (OD_600nm_, 0.4) from a 7-day-old Middlebrook 7H9 (Difco) culture, supplemented with albumin-dextrose-catalase (ADC; Difco), was pelleted by centrifugation at 2500 g for 15 min at 16°C, washed twice with 10% glycerol and finally resuspended in 3 ml of 10% glycerol. Two hundred microliters of the electrocompetent bacilli were mixed with 5 microliter of the control vector pYUB412 (85 ng µl^−1^) or recombinant vector RD1-2F9 (100 ng µl^−1^) and electroporated using the Gene Pulser Xcell Electroporation System (Bio-Rad Pacific, Hong Kong) with settings of 2.5 kV, 25 µF and 1000 Ω. After electroporation, cells were resuspended in 5 ml of 7H9 medium supplemented with ADC, and kept overnight at 37°C. The cells were then pelleted by centrifugation, resuspended in 100 µl of 7H9 medium, and plated on Middlebrook 7H11 medium supplemented with oleic acid-albumin-dextrose-catalase (OADC, Difco), hygromycin (200 µg ml^−1^) and ampicillin (100 µg ml^−1^). After three to four weeks of incubation at 37°C, hygromycin-resistant clones were selected. BCG::RD1-2F9 (BCG::RD1) clones were characterized for secretion of ESAT-6 by immunoblotting using mouse anti-ESAT6 antibody (unpublished data).

### Recombinant ESAT6 protein

Detailed procedures for preparation and characterization of recombinant ESAT6 have been described in our earlier publication [52]. Briefly, E. coli BL21(plysS) transformed with pET23b+ vector (Novagen) carrying esat6 gene of M. tuberculosis was grown to mid-log phase, induced with IPTG (0.4 mM final conc.) for 4 hrs, and the recombinant ESAT6 protein was extracted from the inclusion bodies in 8 M urea. The recombinant ESAT6 protein was then purified by Nickel -nitrilotriacetic acid (Ni-NTA) chromatography, checked for LPS contamination by LAL (limulus amebocyte lysate) tests, and characterized for purity by SDS-PAGE, immunoblotting and N-terminal amino acid sequencing as described previously [52]. The purified and LPS-free recombinant ESAT6 protein was aliquoted and kept at -80°C until further use.

### Bacterial cultures

All mycobacterial strains were grown in 7H9 (Middlebrook, Difco, USA) medium supplemented with 10% ADC and with 0.05% Tween 80 and 0.2% glycerol, and cultures were grown to mid-log phase. Aliquots of the cultures in 20% glycerol were preserved at −80°C and these cryo-preserved stocks were used for infections.

### M. tb infection of mice and estimation of Colony Forming Units (CFU)

Mice were infected with various mycobacterial strains (namely H37Rv, H37RvΔRD1, BCG, or BCG::RD1) via the aerosol route using a Madison aerosol chamber (University of Wisconsin, Madison, WI) with its nebulizer pre calibrated to deposit a total of ∼110 to the lungs of each mouse as previously described [51], [53]. Briefly, mycobacterial stocks recovered from a −80°C freezer were quickly thawed and subjected to light ultra-sonication to obtain a single cell suspension. Fifteen ml of the bacterial cell suspension (10×10^6^ cells per ml) was placed in the nebulizer of the Madison aerosol chamber pre-calibrated to deliver via aerosol route the desired number of CFUs to the lungs of animals placed inside the chamber. A day after the aerosol exposure procedure, three randomly selected mice were sacrificed at various time points and organs were harvested, homogenised in 0.2 µm filtered PBS containing 0.05% Tween 80 and plated onto 7H11 Middlebrook (Difco USA) plates containing 10% oleic acid, albumin, dextrose and catalase (OADC) (Difco USA). Undiluted, ten-fold diluted and one hundred-fold diluted lung and spleen cell homogenates were plated in duplicate on the above 7H11 plates and incubated at 37°C for 21–28 days. Colonies were counted and CFU were estimated. Mice from various groups were euthanized at the indicated time points in various experiments; their organs were harvested for obtaining CFU counts and/or immune cell subpopulations for immunological studies as described under other sub-sections.

### Reagents

Luminex kits were purchased from Millipore and Bio-Rad. GM-CSF and IL-4 were obtained from R&D Biosystems, USA. Purified or fluorescently-conjugated monoclonal antibodies against mouse CD11c (N418), CD11b (M1/70), CD80 (16-10A1), CD86 (GL1), and MHC-II (NIMR-4) were purchased from eBioscience, USA, and fluorescently-conjugated anti-mouse IgG2a (R19-15) was purchased from BD Pharmingen. LPS was obtained from Sigma-Aldrich (L-2654).

### Generation of dendritic cells (DCs)

Mice were euthanized and the femurs were isolated. Bone marrow was flushed out with RPMI-1640 medium using a 2.0 ml syringe (26.5G). The cells were washed twice with PBS and then cultured in complete RPMI-1640 (Gibco, UK) medium supplemented with GM-CSF (40 ng/ml) and IL-4 (10 ng/ml) on 24-well plates (1 million/ml). On the third day, 75% of the medium was replaced with fresh DC culture medium. On day 5, the suspended cells were removed and the loosely adherent cells were collected as immature DCs (CD11c-positive cells were >90%). Flowcytometric analysis by using anti-CD11c, -CD11b, -CD80, -CD86, -MHC class II, and - IgG2a (isotype control) antibodies suggested that >95% of the cells were conventional DCs.

### Bacterial infection of DCs and co-culture with CD4^+^ T cells from OT-II TCR transgenic mice

BM cells were isolated from different mouse strains (C57BL/6, TLR-2^-/-^ and MyD88^-/-^) and differentiated into immature DCs as described above and cultured in 24-well plates (1×10^6^ cells per well). Cells were infected with H37Rv, H37RvΔRD1, BCG or BCG::RD1 (MOI of 1∶10). Similarly, 1×10^6^ DCs were cultured in 24-well plates in the presence or absence of LPS at 1 µg/mL and co-stimulated with ESAT-6 protein at a final concentration of 5 µg/mL, with PBS as the negative control. Supernatants from cells were collected at 24, 48 and 72 hrs for cytokine profiling. For Th1 and Th17 cell differentiation, CD4^+^ T cells (1×10^6^) were purified by MACS method (CD4^+^ T cell isolation beads kit; Miltenyi Biotech, Germany) from OT-II TCR transgenic mice and cultured with immature DCs (1×10^6^) infected with H37Rv, H37RvΔRD1, BCG or BCG::RD1 (MOI of 1∶10) in the presence of ovalbumin (10 µg/ml) peptide (Thermo Scientific, USA) for 72 hours. Then, CD4^+^ T cells were harvested and subjected to intracellular staining for IL-17 and IFN-γ expression.

### Antibiotic treatment of mice

Thirty days post infection, groups of mice were treated with 0.1 g/L rifampicin and 0.1 g/L isoniazid (Sigma-Aldrich, St. Louis, MO, USA) administered in the drinking water (changed daily) for 4 weeks. *M. tuberculosis*-infected control mice received plain drinking water. A control group of infected mice was sacrificed at the start of treatment (early control group). A second group of infected but untreated mice was sacrificed 4 weeks after therapy was initiated (late control group).

### Isolation of lymphocytes from infected animals

Lungs from infected or uninfected animals were harvested and washed by swirling in PBS. They were opened up by cutting longitudinally and then cut into ∼0.5-cm pieces. These lung pieces were agitated in 25 ml of extraction buffer (PBS, 3% FCS, 1 mM dithiothreitol, 1 mM EDTA) for 30 min at 37°C. This slurry was passed through a loosely packed nylon wool column to remove the aggregates. The filtrate was layered on a discontinuous Percoll gradient (Amersham Pharmacia Biotech, USA). This gradient was then centrifuged at 900 × *g* for 20 minutes. Cells at the interface of the 40/70% layer were collected and washed in staining buffer (PBS, 3% FCS). Cells were cultured for intracellular staining as described below. Bronchoalveolar lavage (BAL) fluid was collected from lungs by intratrachial infusion of PBS and cell-free BAL was used for cytokine assay [54].

### [^3^H]-Thymidine incorporation assay of splenocytes

Spleens were isolated from sixteen randomly selected C57BL/6 mice of our colony or CSA-immunized (100 µg/ml in 200 µl incomplete Freund's adjuvant) mice. Spleens were macerated by frosted slides in complete RPMI 1640 (Gibco, Invitrogen, UK) and made into a single cell suspension. Red blood cells (RBCs) were lysed with RBC cell lysis buffer and washed with complete RPMI 1640. Splenocytes were counted and plated at 0.1×10^6^ cells/well in 96-well plates and stimulated with different concentrations of M. tb complete soluble antigen (CSA). Cells were cultured for 48 hours and then pulsed with tritiated thymidine (^3^H-TdR, 1.0 µCi per well; Amersham Biosciences UK) before measuring incorporation of ^3^H-TdR by means of a cell harvester and liquid scintillation counter 16 hours later (Wallac Trilux, Perkin Elmer, UK).

### Intracellular cytokine staining

For intracellular cytokine staining, cells were treated with 50 ng/ml phorbol myristate acetate (PMA) and 500 ng/ml ionomycin in the presence of 10 µg/ml brefeldin A (Sigma-Aldrich or eBiosciences, USA) added during the last 6 h of culture. Cells were washed twice with PBS and resuspended in a permeabilization buffer (Cytofix/Cytoperm kit; BD), and stained with the following fluorescently conjugated monoclonal antibodies: anti-CD4 (clone: GK1.5)-APC, anti-IFN-γ (clone: XMG1.2)-FITC, and anti-IL-17 (clone: 17B7)-PE (all from eBiosciences, USA). Fluorescence intensity was measured by flow cytometry (FACS Calibur; BD) and data were analysed with FlowJo (Tree star, USA).

### q-PCR analysis

Bone marrow derived DC were isolated and infected with different bacterial strains (H37Rv, H37RvΔRD1, BCG and BCG::RD1) as described above and cultured for 24 hours for RNA isolation. Total RNA, including miRNAs was isolated by miRNAeasy isolation kit (QIAGEN, Germany) according to the manufacturer's instructions. Real-time quantitative RT-PCR analysis was performed using BioRad Real-Time thermal cycler (BioRad, USA) and miRCURY LNA universal reverse transcriptase microRNA PCR SYBR green master mix (EXIQON, Denmark) for miRNA amplification and IQ BioRad SYBER green master mix (BioRad, USA) for IL-6 expression, respectively. cDNA was synthesized by the miRCURY LNA universal reverse transcriptase microRNA cDNA synthesis kit (EXIQON) and the reaction was set up according to the manufacturer's protocol. For amplification of miR146a, LNA PCR miR146a and reference 5S rRNA primer sets were used and the reaction was set up as recommended by EXIQON. The relative expression level of miRNAs was normalized to that of internal control 5S rRNA by using 2-ΔΔCt cycle threshold method. Furthermore, for amplification of IL-6 or IL-22, cDNA was synthesized by Omniscript RT kit (QIAGEN) using oligodT primers (Fermentas, Maryland, USA). For IL-6 mRNA expression analysis primer sequences were IL-6F, 5′-TGGAGTCACAGAAGGAGTGGCTAAG-3′ and IL-6R, 5′- TCTGACCACAGTGAGGAATGTCCAC-3′, and control GAPDH-F, 5′- CGTCCCGTAGACAAAATGGT-3′ and GAPDH-R, 5′- TTGATGGCAACAATCTCCAC-3′ and for IL-22 mRNA expression analysis primer sequences were IL-22F 5′-GTGACGACCAGAACATCCAG-3′ and IL22R 5′-ATCTCTCCGCTCTCTCCAAG-3′. Data were normalized by the level of GAPDH expression in samples as described above.

### Knock-down of miR146a using anti-miR146a

For transfection of anti-miR146a and scramble control (EXIQON) into DCs, cells were transfected at day 4 of culture in antibiotic free media using Lipofectamine (Invitrogen, UK) reagents and at day 5 cells were infected with H37Rv, H37RvΔRD1, BCG or BCG::RD1. After 24 hours of bacterial infection, cells were harvested for RNA preparation and analyzed for miR146a and IL-6 expression by quantitative real-time PCR as described above.

### Detection of cytokines

Cytokines in the culture supernatant of DCs were assayed by a Luminex microbead- based multiplexed assay using commercially available kits according to the manufacturer's protocol (Milliplex kit, Millipore and BioPlex, Bio-Rad, USA).

### Statistical analysis

All data were derived from at least three independent experiments. Statistical analyses were conducted using SPSS software and values were presented as mean±SD. Significant differences between the groups were determined by ANOVA followed by Tukey's multiple comparison test (SPSS software). A value of p<0.05 was accepted as an indication of statistical significance.

### Accession numbers of proteins mentioned in the manuscript

CFP-10: 10 kDa culture filtrate protein [Mycobacterium tuberculosis H37Rv]. ACCESSION ACH88465.

ESAT-6: early secreted antigenic target 6 kDa [Mycobacterium tuberculosis H37Rv]. ACCESSION AAC83446.
